# Characteristics and expression patterns of six α-galactosidases in cucumber (*Cucumis sativus* L.)

**DOI:** 10.1371/journal.pone.0244714

**Published:** 2021-01-12

**Authors:** Zhi-ping Zhang, Yan-cheng Liu, Hai-bo Dai, Min-min Miao

**Affiliations:** School of Horticulture and Plant Protection, Yangzhou University, Yangzhou, People’s Republic of China; Assam Agricultural University Faculty of Agriculture, INDIA

## Abstract

Six putative α-galactosidase genes (α-Gals), three acid forms (*CsGAL*1, *CsGAL*2, *CsGAL*3) and three alkaline forms (*CsAGA*1, *CsAGA*2, *CsAGAL*3), were found in the cucumber genome. It is interesting to know the expression pattern and possible function of these α-Gals in the cucumber plant since it is a stachyose-translocating species. In this study, full-length cDNAs of six α-Gals were cloned and heterologously expressed. The result showed that all recombinant proteins revealed acid or alkaline α-Gal activities with different substrate specificities and pH or temperature responding curves, indicating their distinct roles in cucumber plants. Phylogenetic analysis of collected α-Gal amino acid sequences from different plants indicated that the ancestor of both acid and alkaline α-Gals existed before monocots and dicots separated. Generally, six α-Gal genes are universally expressed in different cucumber organs. *CsGAL2* highly expressed in fasting-growing leaves, fruits and germinating seeds; *CsGAL3* mainly distributes in vacuoles and significantly expressed in cucumber fruits, senescent leaves and seeds during late stage germination; The expression of *CsAGA1* increased from leaf 1 to leaf 3 (sink leaves) and then declined from leaf 4 to leaf 7 (source leaves), and this isoform also highly expressed in male flowers and germinating seeds at early stage; *CsAGA2* significantly expressed in cucumber leaves and female flowers; *CsAGA3* is localized in plastids and also actively expressed in senescent leaves and germinating seeds; The role of *CsGAL1* in cucumber plants is now unclear since its expression was relatively low in all organs. According to their expression patterns, subcellular localizations and previously reported functions of these isoforms in other plants, combining the data of soluble sugars contents in different tissues, the possible functions of these α-Gals were discussed.

## Introduction

Alpha-galactosidases (α-Gals, EC3.2.1.22), also known as α-D-galactoside galactohydrolase or melibiase, is an exoglycosidase that hydrolyses the terminal nonreducing α-galactosyl moieties from galacto-oligosaccharides, polymeric galactomannans, glycolipids or glycoproteins [1, 2]. Protein structure and cluster analyses have indicated that α-Gals can be classified mainly into the glycoside hydrolase families GH4, GH27, GH36, GH57, GH97 and GH110 (http://www.cazy.org/). Basically, α-Gals from eukaryotes are grouped into GH 27, while procaryotic α-Gals are mostly classified into GH36 [3]. In human beings, α-Gals cleaves the terminal α-galactose residue from glycolipids and glycoproteins to avoid Fabry disease caused by incomplete degradation of carbohydrates [4]. In microorganisms, α-Gals allow bacteria or fungi to survive under environments where galactoside residues are available [5].

Alpha-Gals also widely distribute in different organs of high plants, including seeds, leaves and fruits [6–8]. According to their activity in response to pH, α-Gals were classified into two families, acid α-Gals and alkaline α-Gals. Normally, there are multiple copies of both acid α-Gals and alkaline α-Gals in plant genomes. According to their subcellular localization, acid α-Gals were further divided into two groups, apoplastic α-Gals and vacuolar α-Gals [9, 10]. The signal peptide of apoplastic α-Gals involve in secretory pathway, which may target them into the extracellular space to hydrolyze cell wall-associated galactomannan during seed maturation or germination [11]. Apoplastic α-Gals also fulfill an important role in leaf development, fruit ripening or aerenchyma formation by functioning in cell wall loosening or cell wall expansion [8, 12, 13]. Comparing with apoplastic α-Gals, the research about vacuolar α-Gals is rare. It is reported a cucumber vacuolar α-Gal is responsible for consuming raffinose family oligosaccharides (RFOs) in vacuole after stress removal [9]. Alkaline α-Gals, with an optimal pH of 7–8, was initially reported in *Cucurbita pepo* leaves [14]. Unlike their acid isoforms, which raffinose was the preferred substrate, early reported alkaline α-Gals show high activity for stachyose and little affinity toward raffinose [15]. Gao and Schaffer (1999) reported a novel alkaline α-Gal from *Cucumis melo* fruit with a high substrate affinity for raffinose [16]. Several evidences have indicated that alkaline α-Gals, rather than acid α-Gals, are responsible for RFOs unloading into fruits and other sink organs of RFOs translocated plants [16–20]. Carmi et al (2003) first cloned two alkaline α- Gal cDNAs from *C*. *melo* and found that their amino acid sequences are distinct from acid forms but have high sequence homology with a group of seed imbibitions proteins (SIPs), indicating SIPs are alkaline α-Gals actually [21]. This founding and several other reports suggested that alkaline α-Gals may be responsible for the rapidly metabolism of RFOs during seed germination [7, 22]. In addition, the accumulation of alkaline α-Gals also be found in leaves or shoots under starving or drought stress [6, 23]. Up to date, alkaline α-Gals were only found in high plants.

Stachyose and raffinose are major RFOs translocated into the cucumber phloem. These oligosaccharides are rapidly catabolized by α-Gals when they arrive at fruits, young leaves and other sink tissues [24]. In addition, RFOs are synthesized in mature cucumber seeds, which were rapidly decomposed by α-Gals after imbibition [25]. RFOs also accumulate in different cucumber organs under cold stress and then are consumed by α-Gals after stress removal [9, 26]. In the cucumber genome, there are six putative α-Gal genes, three acid α- Gal genes (*CsGAL*1, *CsGAL*2, and *CsGAL*3) and three alkaline α-Gal genes *(CsAGA*1, *CsAGA*2, and *CsAGA*3). However, the exact roles of these α-Gals in the biochemical process mentioned above are still unclear. As a typical RFOs translocated species, the functions of different α-Gals of cucumber should be more complex and interesting than those of sucrose translocated species. In this study, full-length cDNA of six putative α-Gal genes were *in vitro* expressed and characterized, and their expression pattern during leave development and seed germination were investigated. Furthermore, α-Gals are widely used in food and feed industry to eliminate anti-nutritional factors and in medicinal field for treatment of Fabry disease or blood enzymatic conversion of A and B to O type [27, 28]. Therefore, the illumination of enzyme properties of cucumber α-Gals was also beneficial for accelerating industrial application of plant originated α-Gals.

## Materials and methods

### Cloning of full-length cDNA of six cucumber α-Gals

Several acid and alkaline α-Gal cDNAs were selected from NCBI and primers for amplifying cDNA fragments were designed in the highly conservative sequence regions (S1 and S2 Figs). Traditional RT-PCR was adapted to clone these fragments using polyT as RT primers. Full-length cDNA of each α-Gal gene were obtained by Rapid Amplification of cDNA Ends technology (RACE) using SMARTer^TM^ RACE cDNA Amplification Kit (Clontech, TaKaRa, Dalian, China). All primers for this purpose were list in the S1 Table in S1 File.

### *In vitro* expression and purification of six cucumber α-Gals

Six cucumber α-Gals were expressed in the s/f 9 insect cells with the BD BaculoGold™ Baculovirus Expression System. Basically, coding regions of six cucumber α-Gal cDNAs were cloned into the multiple cloning site of pAcHLT serial vectors (Pharmingen, BD Biosciences, New Jersey, USA) by standard restriction digest and ligation reactions, the primers and restriction enzymes used for cloning were listed in S2 Table in S1 File. The recombinant proteins were expressed in and purified using 6×His Baculovirus Expression and Purification Kit (Pharmingen), according to the manufacturer`s instruction. 6×His tag were removed from purified proteins with the proteolytic thrombin cleavage (provided by the same kit). The molecular weight of recombinant proteins was evaluated by SDS-PAGE together with Molecular-mass standards (Pharmacia, Uppsala, Sweden).

### Experimental setup for enzyme property assay

The substrate specificity of purified α-Gals was assayed with artificial substrate p-nitrophenyl a-galactopyranoside (pNPG) and natural substrate raffinose and stachyose. Km values for these substrates were determined by Lineweaver-Burk plots. The optimum pH for each purified enzyme was determined using 5 mm pNPG as a substrate in 100 mM McIlvaine buffer over a pH range of 3.5 to 6.5, HEPES buffer from pH 7.0–8.0, and Glycine-NaOH buffer from 8.5–9.0, all at 37°C. The effect of temperature on the activity of the purified enzymes was measured from 20°C to 50°C, at pH 5.0 (for acid α-Gals) or pH 7.5 (for alkaline α-Gals).

### Sequence alignment and phylogenetic tree analysis of α-galactosidase

46 putative amino acid sequences of the published α-galactosidase gene in 21 higher plants species were retrieved and downloaded from NCBI (https://www.ncbi.nlm.nih.gov/, S3 Table in S1 File). The amino acid sequence homology alignment of these putative proteins were performed with DNAMAN 6.0 software. For phylogenetic analysis, sequences were formatted and aligned by using Clustal X 1.83, and visualisation was performed with MEGA 7.0 programs.

### Plant growth and sample collection

Cucumber (*Cucumis sativus* L. var. *Jinyou 35*, Tianjin Cucumber Institute, China, monecious, North China type) plants were grown in 40 ×40 cm plastic pots in a growth chamber according to our published paper [29]. All cucumber organs were sampled at 19 leaf stage. To obtain different cucumber organs, leaves, fruits (8 d after anthesis), stems, female flowers (with ovary together) and male flowers (at anthesis) from 9^th^ to 11^th^ node were collected; To study the function of α-Gals in leaf development to senescence, 1^st^ to 7^th^ leaf, the 9^th^ leaf (matural leaf), 14^th^ leaf (senescence leaf 1) and 15^th^ leaf (senescence leaf 2) were collected (Fig 4). The 14^th^ leaf and 15^th^ leaf were defined as senescence leaves since they were the first and second leaf beginning to reveal foliage yellowing.

### Seed germination treatment

Cucumber seeds were immersed in water for 4 hours at room temperature, and then placed on the water-saturated paper towels for germination at 25°C. The germinating seeds were assayed for both short and long periods. For short period assay, seeds were sampled 0 h, 4 h, 8 h, 12 h, 16 h, 20 h and 24h after imbibition. For long period assay, seed samples were continuously collected 1 d, 2 d, 3 d, 4 d and 5 d after imbibition.

### Sample preservation

All organ tissues were weighed and immediately frozen in liquid nitrogen and then stored at -80°C.

### Total RNA isolation and expression analysis of α-galactosidases

Total RNA was extracted from different cucumber organs using RNeasy Plant Mini Kit (QIAGEN, Shanghai, China) kit. The expression levels of α-galactosidases were analyzed by RT-qPCR using the One Step SYBR PrimeScript RT-PCR kit (TaKaRa, Dalian, China) on an CFX96 Touch™ Real-Time PCR Detection System (Bio-Rad, USA). The specific primers of *CsGAL1*, *CsGAL2*, and *CsGAL3*, *CsAGA1*, *CsAGA2*, and *CsAGA3* were designed using Primer premier5.0 software and listed in S4 Table in S1 File. Real-time reverse transcription (RT)-PCR was performed using the One Step SYBR PrimeScript RT-PCR Kit (TaKaRa) following manufacturer’s instruction. The real-time PCR was carried out according to the following protocol: 2 min at 94°C, followed by 39 cycles of 94°C for 15 s, 60°C for 15 s and 72°C for 30 s. The equation ratio 2^-△△Ct^ was applied to calculate the relative expression level of the six α-galactosidases encoding gene using the cucumber 18S rRNA gene internal control [30]. The data were analyzed by the Bio-Rad CFX Manager software.

### Sugar analysis and α- Gal activity determination

Stachyose, raffinose, sucrose, glucose and fructose were extracted and analyzed with HPLC as described by our published paper [29]. The α- Gal extraction and activity assay was according to Miao et al (2007) if using pNPG as substrate or Wang et al (2016) if using raffinose and stachyose as substrates, except the synthesized galactose was determined with HPLC [29, 31]. The enzyme activity was converted to nKat protein mg^-1^.

### Statistical analyses

Variance analysis and significant difference tests were carried out to determine differences among means by one-way ANOVA. Excel 2010 and OriginPro 8.0 were used to process the data and map.

## Results

### *In vitro* expression and characterization of six cucumber α-Gals

**Fig 1:**
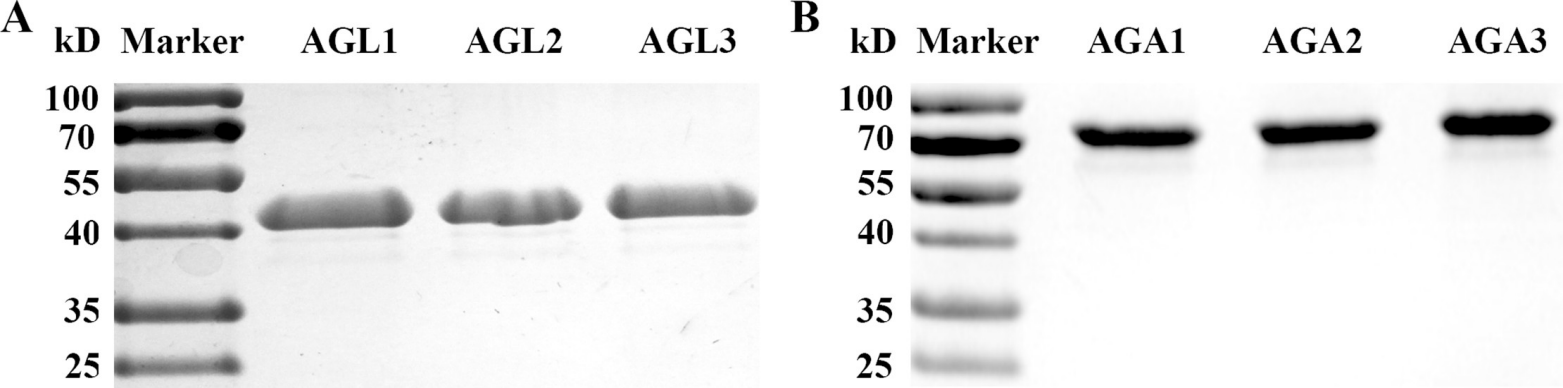
Purified cucumber α-Gals from s/f 9 insect cells. The recombinant cucumber α-Gals were heterologously expressed in the s/f 9 insect cells. Proteins were separated by 10% SDS-PAGE, and analysis using gel imaging systems camera.

Full-length cDNA of six cucumber α-Gal cDNAs were obtained by RACE. Some characteristics of these genes, include gene constructions and deduced protein properties were listed in Table 1. To confirm that the six putative cucumber α- Gal genes in fact encode for acid or alkaline α-Gals, they were heterologously expressed in insect cells. The eukaryotic expression system was selected since glycosylation may influence the biological activity of acid α-Gals [32]. The six recombinant proteins were separated by SDS-PAGE. The result showed each recombinant protein has single protein bands, and the size of three acid α-Gal was about 45 kD, and that of alkaline α-Gal was about 80 kD (Fig 1), which agreed almost exactly with that of calculated from the deduced amino acid sequences (Table 1).

**Table 1:** Molecular biological information of cucumber α-galactosidases.

Name	GenBank accession number (Nucleotide_id)	Exon Number /intron Number	cDNA Full-Length (bp)	5`UTR/ 3`UTR Length (bp)	CDS Length (bp)	GenBank accession number (protein_id)	Protein aa number	Protein Molecular Weigh (KDa)	Isoelectric Point of Protein (pI)
CsGAL1	DQ320569.1	15/14	1561	115/204	1242	ABC55266.1	413	45.70	5.69
CsGAL2	DQ361277.1	15/14	1634	184/184	1266	ABC88435.1	421	46.64	7.55
CsGAL3	NM_001305751.1	15/14	1798	137/368	1293	AEQ94270.1	430	47.91	5.71
CsAGA1	DQ157703.2	13/12	2685	219/204	2262	AAZ81424.2	753	82.79	6.05
CsAGA2	DQ395330.2	14/13	2844	249/276	2319	ABD52008.2	772	84.49	5.65
CsAGA3	JQ327828.1	6/5	2804	289/163	2352	AFA34435.1	783	86.10	5.29

**Fig 2:**
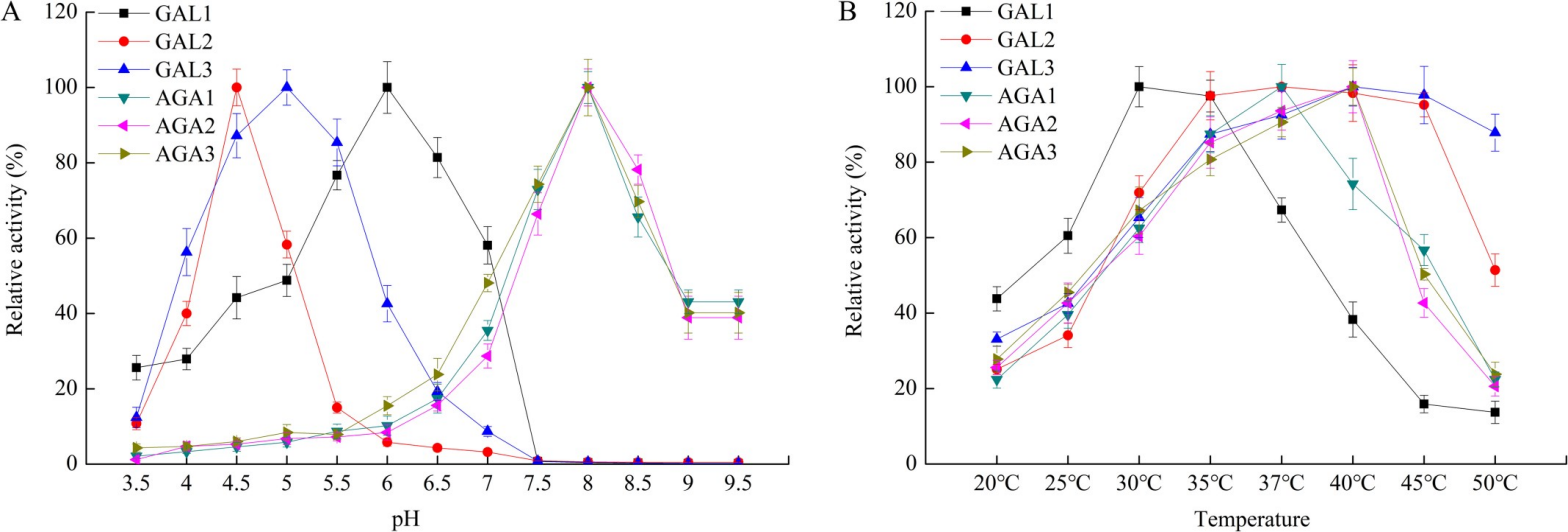
Effects of pH value and temperature on activity of cucumber α-Gals. The activity of recombinant cucumber α-Gals were measured as described under “Materials and Methods”. A, the effect of pH value on the activity of recombinant cucumber α-Gals. B, the effect of temperature on the activity of recombinant cucumber α-Gals. All reactions were performed in three biological repetition and three technical repetition, the average values were calculated, and vertical bars represent standard errors.

The effects of pH and temperature on the activity of six cucumber α-Gals were evaluated with pNPG as substrate. As shown in Fig 2A, among three acid α- Gal genes, CsGAL1, CsGAL2 and CsGAL3 were most active at pH 6.0, 4.5 and 5.0, respectively. Comparing to CsGAL2, CsGAL1 and CsGAL3 have relative wider activity pH ranges. All three alkaline α- Gals have similar optimum pH of 8.0, and high activity was maintained from pH 7.5 to pH 8.5 (relative enzyme activity >50%). The temperature response curve showed that the maximal activity was at 30°C of CsGAL1, 37°C of CsGAL2 and CsAGA1, and 40°C of CsGAL3, CsAGA2 and CsAGA3, respectively (Fig 2B).

The substrate specificities of α-Gals were investigated with pNPG, raffinose and stachyose. Among three substrates, all α-Gals have the highest affinity with pNPG. Three acid α-Gals hydrolyzed raffinose more efficiently than stachyose, while CsAGA2 and CsAGA3 have significantly higher activity with stachyose than raffinose. Unlike these two isoforms, the substrate specificity of CsAGA1 to stachyose is only a little higher than that to raffinose (Table 2).

**Table 2:** Characteristics of in vitro expressed cucumber α-galactosidases.

Substrate	α-galactosidases	Km (mM)	Activity (μmol.mg-1 protein. min-1)
pNPG	GAL 1	0.89±0.12 c	8.91±1.59 a
	GAL 2	1.35±0.23 b	4.23±0.74 c
	GAL 3	0.99±0.10 bc	1.56±0.65 d
	AGA 1	1.81±0.39 a	1.42±0.27 d
	AGA 2	2.21±0.28 a	6.84±0.54 b
	AGA 3	1.24±0.17 bc	5.32±1.46 bc
Raffinose	GAL 1	4.71±0.85 cd	0.89±0.15 a
	GAL 2	5.63±0.67 c	0.71±0.27 ab
	GAL 3	3.24±0.70 cd	0.92±0.19 a
	AGA 1	2.08±0.51 d	0.48±0.11 bc
	AGA 2	30.4±2.85 a	0.21±0.08 c
	AGA 3	22.47±1.87 b	0.25±0.05 c
Stachyose	GAL 1	12.3±1.92 b	0.47±0.07 c
	GAL 2	11.85±2.05 b	0.23±0.05 c
	GAL 3	18.92±2.56 a	0.17±0.06 c
	AGA 1	3.76±0.89 c	0.64±0.03 c
	AGA 2	5.23±1.23 c	2.2±0.60 b
	AGA 3	3.26±0.76 c	4.88±0.92 a

Note: The same lower case letters indicate that there is no significant difference in Km or activity of different kinds of α-galactosidases under the same reaction substrate.

### Phylogenetic analysis of six cucumber α-Gals

**Fig 3:**
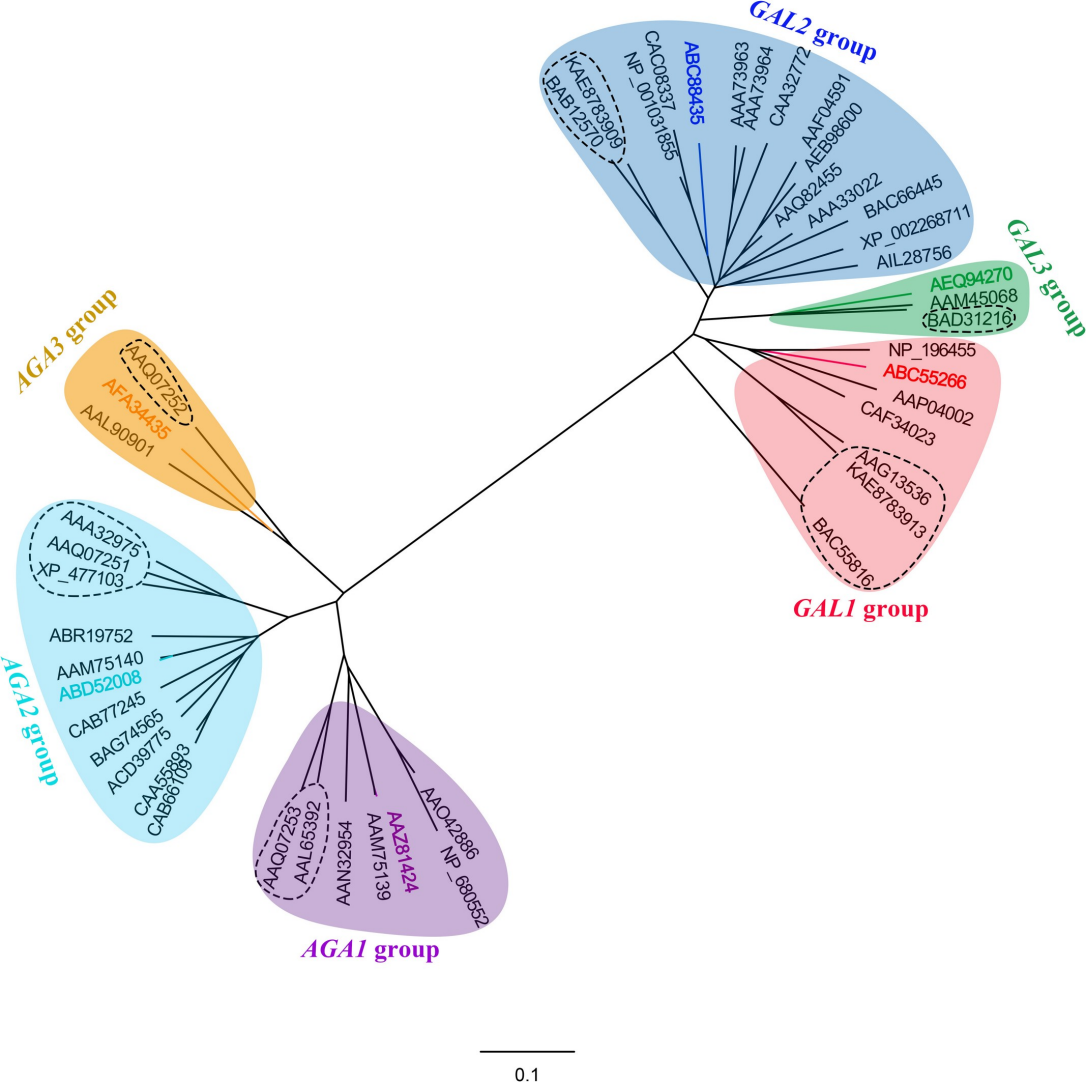
Phylogenetic relationships among 46 exemplars of α-galactosidase from 21 plant species. Phylogenetic tree was carried out based on amino acid sequence of α-galactosidase by the Maximum likelihood (ML) and neighbor-joining method using MEGA 7.0 software. Accession numbers and the detailed information corresponding to the α-galactosidase proteins were list in the S3 Table in S1 File. 46 α-galactosidase genes were grouped into two major clusters, the acidic form and the alkaline form, and the monocotyledons are denoted with the dotted box, others are dicotyledons. Cucumber α-galactosidases were divided into 6 sub-clades and labeled with different colors as follows: *CsGAL1* (DQ320569), Orange; *CsGAL2* (DQ361277), Brown; *CsGAL3* (NP_001292680), Pink; *CsAGA1* (AAZ81424), Purple; *CsAGA2* (ABD52008), Blue; *CsAGA3* (NP_001267640), Green.

Amino acid sequences of 46 putative α-Gals, including six cucumber α-Gals, were collected from NCBI and the phylogenetic relationship of them was analyzed. As shown in Fig 3, total plant α-Gals were divided into two subfamilies, acid and alkaline. Both acid α-Gals and alkaline α-Gals were further classified into three groups, named GAL1, GAL2, GAL3, AGA1, AGA2 and AGA3, each group contain one of the six cucumber α- Gal. Possible functions and papers reported selected α-Gals were recapitulated in the S3 Table in S1 File. In addition, we found that some α-Gals belong to the same group have similar properties, several examples were listed as follows: CsAGA1 and CmAGA1 exhibit very high homology of their amino acid sequence and also have similar substrate specificity, *i*.*e*., similar affinity to raffinose and stachyose; All α-Gals belong to the GAL3 group have a C-terminal oligopeptide extension which was defined as a non-sequence-specific vacuolar sorting determinant of plant galactan: galactan galactosyltransferase and acid galactosidase [10], indicating the vacuolar subcellular localization of this group of α-Gals; Both CsAGA3 and AAL90901 belong to the AGA3 group and were found to distribute in chloroplasts [6, 9], *etc*. However, it is also common that some α-Gals classified into different groups revealed similar functions, while other α-Gals with different biological roles belong to the same group (Fig 3).

### Expression patterns of cucumber α-Gals in different organs

**Fig 4:**
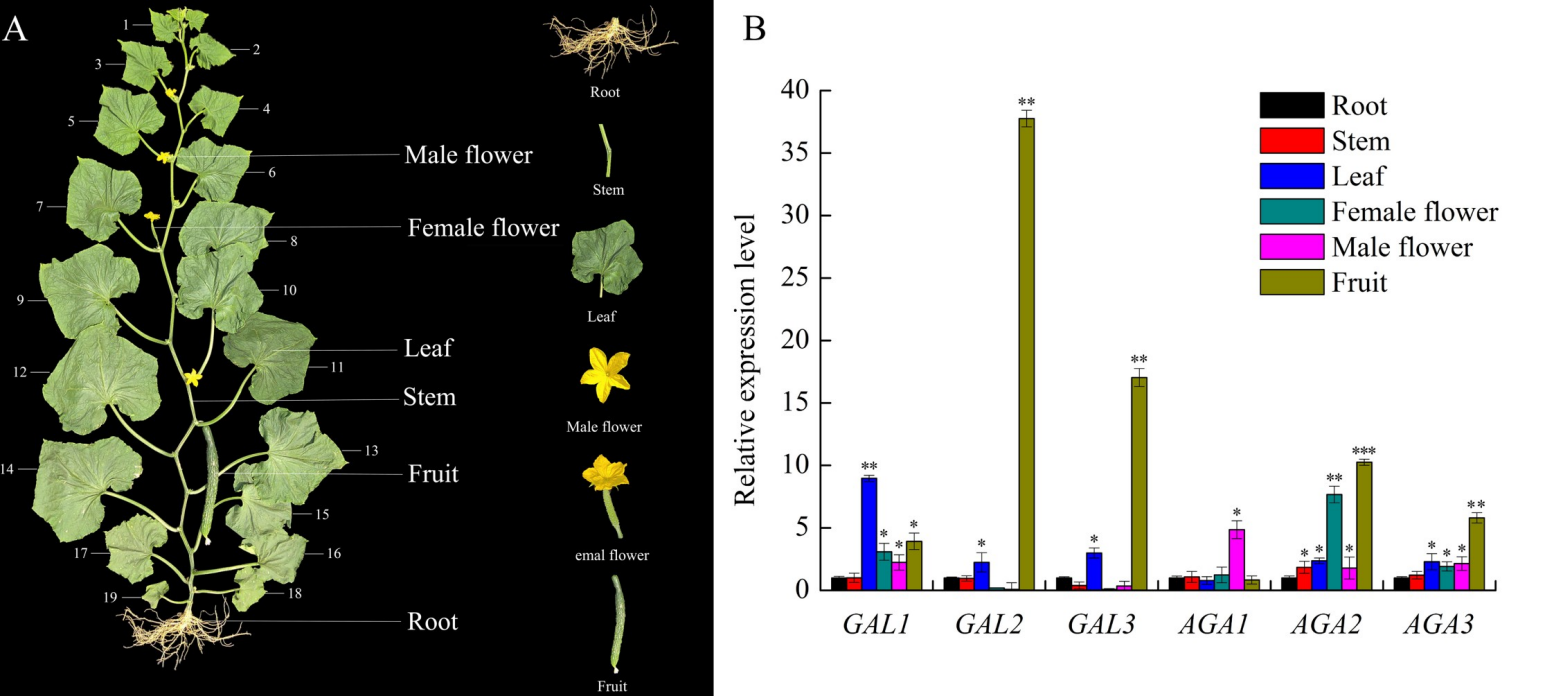
Expression analysis of α-galactosidases in the different cucumber organs. **(**A) Cucumber plants with 19 leaves were used in the experiment. Root, stem between 9^th^ to ^11^th node, the ^11^th mature leaves, female flower, male flower and fruit at the ^11^th node at 8 days after anthesis were sampled. (B) RT-PCR efficiency was used to calculate the expression of target genes relative to the expression of cucumber *18S* RNA gene, and relative amounts were normalized with respect to the expression of each forms of α-galactosidasegene in cucumber root. All reactions were performed in three biological repetition and three technical repetition, the average values were calculated, and vertical bars represent standard errors. Different letters indicate significant differences among various types of α-galactosidase in the same cucumber organ at P<0.05.

As shown in Fig 4, expressions of all six α-Gal genes were found in sampled cucumber organs, including root, stem, leaf, female flower, male flower and fruit, indicating these genes exist almost universal in cucumber plants. Basically, all α-Gal genes were expressed at low level in roots and stems. Among six α-Gal genes, *CsGAL2*, *CsGAL3*, *CsAGA2* and *CsAGA3* are highly expressed in fruits, *CsGAL1* is the predominant isoform in leaves, while *CsAGA1* and *CsAGA2* are significantly expressed in male flowers and female flowers, respectively. These results indicate there should be significant catabolic activity of α-1, 6-linked galactosyl residues in these organs. In addition, the high expression of these α-Gal genes suggested they may perform important roles in certain organs.

### Changes of expression levels of six α-Gal genes and related soluble sugar contents during cucumber leaf development and senescence

**Fig 5:**
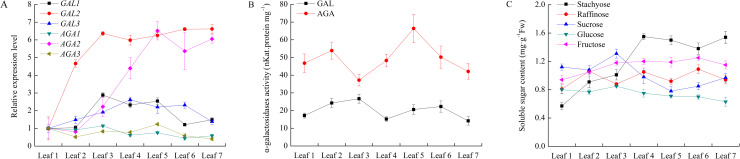
Change of relative expression level and enzyme activity of α-galactosidase and soluble sugar content during cucumber leaf development. Cucumber plants at 19 leaf stage were selected. Leaf 1: the first unfold leaf from the top of plant. Leaf 2–7: from top to bottom, followed leaf 1, defined as Leaf 2, 3, 4, 5, 6 and 7respectively. (A) Expression of α-galactosidases in cucumber leaves during development. The relative amounts were normalized with respect to the expression of each gene in Leaf1. (B) Enzyme activity of acid and alkaline α-galactosidases of cucumber leaves during development. (C) Chang of soluble sugar content during cucumber leaves development. All reactions were performed in three biological repetition and three technical repetition, the average values were calculated, and vertical bars represent standard errors.

During cucumber leaf development from leaf 1 to leaf 7, among six α-Gal genes, only *CsGAL2* and *CsAGA2* revealed significant incremental expression patterns, arriving at the plateau at leaf 3 for *CsGAL2* and leaf 5 for *CsAGA2*. The expression of *CsGAL1* and *CsGAL3* was high at leaf 3 and leaf 4 respectively. The mRNA level *CsAGA1* sightly increased from leaf 1 to leaf 3 and then delined. The *CsAGA3* transcipt level did not change significantly among leaves at different developmental stages (Fig 5). The activities of both acid and alkaline α-Gals fluctuated duirng leaf development (Fig 5). From cucumber leaf 1 to leaf 7, contents of stachyose and frucose increased and become two prevailing soluble sugars in the leaf 7. Sucrose content decreased while glucose content remained little change and at relatively low level during this period (Fig 5).

**Fig 6:**
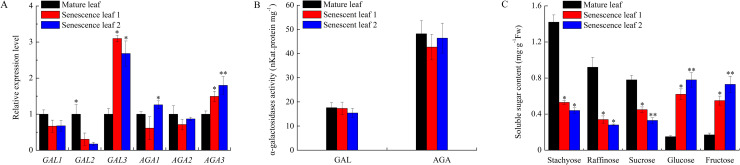
Change of relative expression level and enzyme activity of α-galactosidase and soluble sugar content during cucumber leaf senescence. (A) Expression of α-galactosidases in cucumber leaves during senescence. The relative amounts were normalized with respect to the expression of each gene in mature leaf of cucumber with 19 leaves. Mature leaf: leaf 11; Senescence leaf 1: leaf 14; Senescence leaf 2: leaf 15. (B) Enzyme activity of acid and alkaline α-galactosidases in cucumber leaves during senescence. (C) Chang of soluble sugar content in cucumber leaves during senescence. All reactions were performed in three biological repetition and three technical repetition, the average values were calculated, and vertical bars represent standard errors. Asterisks “*” indicate significant differences between mature and senescent leaves.

Comparing to cucumber mature leaves, the expression level of *CsGAL*2 decreased while *CsGAL*3 and CsAGA3 increased in senescent leaves. No significant differences of acid and alkaline α-Gal activities were observed between mature and senescent leaves. Leaf senescence also effected the sugar content significantly. The contents of stachyose, raffinose and sucrose were obviously lower while the level of glucose and fructose were much higher in senescent leaves than those in mature leaves (Fig 6).

### Change of expression levels of six α-Gal genes and related soluble sugar contents during seed germination

**Fig 7:**
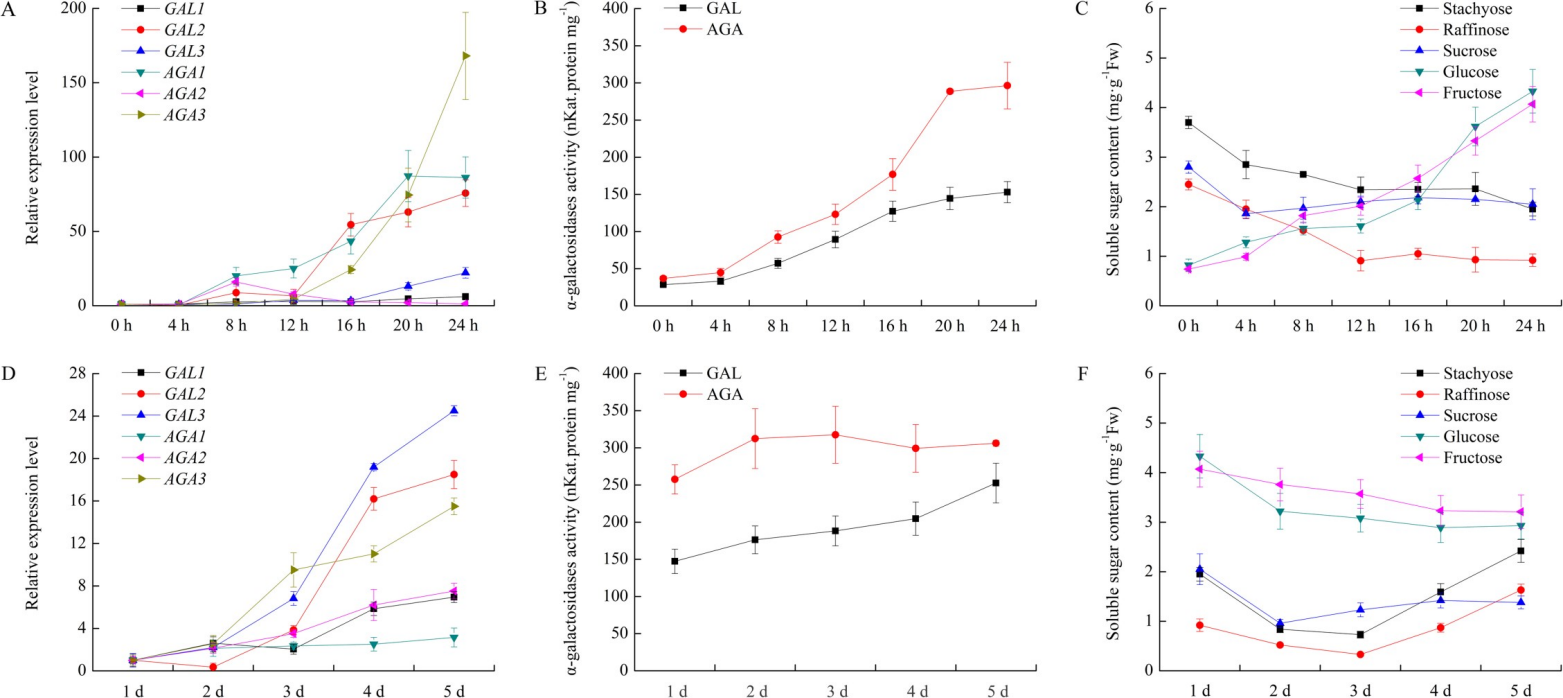
Change of relative expression level and enzyme activity of α-galactosidase and soluble sugar content during cucumber seeds germination. (A) Expression of α-galactosidases during cucumber seeds germination within 24 hours. The relative amounts were normalized with respect to the expression of each gene in seeds at 0h seed germination. (B) Enzyme activity of acid and alkaline α-galactosidases during cucumber seeds germination within 24 hours. (C) Chang of soluble sugar content during cucumber seeds germination within 24 hours. (D) Expression of α-galactosidases in the later stage of cucumber seed germination. The relative amounts were normalized with respect to the expression of each gene in seeds at 24h seed germination. (E) Enzyme activity of acid and alkaline α-galactosidases in the later stage of cucumber seed germination. (F) Chang of soluble sugar content in the later stage of cucumber seed germination. All reactions were performed in three biological repetition and three technical repetition, the average values were calculated, and vertical bars represent standard errors.

We investigated the levels of different α-Gal mRNAs and related soluble sugars after seed imbibition. In a short period (24 h), the expression of *CsGAL2*, *CsAGA1* and *CsAGA3* increased rapidly after 12 h, while mRNA level of *CsGAL3* only slightly raised after 16 h. Little fluctuation of the expression quantities of other two α-Gal mRNAs, *CsGAL1* and *CsAGA2*, were observed. In the same period, the concentrations of stachyose, raffinose and sucrose declined remarkably during the first 12 h after imbibition, indicating rapid metabolism of these sugars during this period. Conversely, the contents of glucose and fructose increased significantly in the 1^st^ day after imbibition. In a long period (1d to 5 d), the expression of all six α-Gal genes showed upward trends, among which *CsGAL2*, *CsGAL3* and *CsAGA3* increased more remarkably than other three α-Gal mRNAs. Glucose and fructose were two predominant sugars in germinating seed, although their concentrations decreased slightly during this period. Interestingly, levels of stachyose, raffinose and sucrose declined from 1 d to 2 d, and then increased from 4 d to 5 d after germination (Fig 7). In both short and long germinatin periods, the acid and alkaline α-Gal activities progressively increased.

## Discussion

All six heterologously expressed cucumber putative α-Gals revealed enzyme activities to artificial substrate pNPG and natural substrates raffinose and stachyose, indicating they are actually α-Gals. Basically, CsGALs exhibited higher affinity to raffinose than that to stachyose, while CsAGA2 and CsAGA3 showed preferred activity with stachyose, which agree with previous studies [33]. CsAGA1, which is highly homologous with CmAGA1 in melon [21], also revealed significant activity to both raffinose and stachyose. Unlike CsAGAs, which had similar response to pH, the optimum pH of three CsGALs varied in a wide range. pH of apoplastic space or vacuole in plant cells, where acid Gals are mainly located, is regulated by biotic or abiotic stress [34, 35]. Thus, diverse optimum pH of CsGALs could facilitate them to fulfill functions under different physiological status. The response of enzyme activity to the temperature change were also different among cucumber α-Gals (optimum temperature from 30°C to 40°C). The physiological significance of this optimum temperature diversity is now not clear. It was found that three acid *Oryza sativa* α-Gals have similar optimum pH (5) and temperature (45°C) [36]. However, we noticed that the authors expressed these α-Gals in *E*. *coli*, the lack of post translation modification of these acid α-Gals in the prokaryotic expression system may affect the enzyme response to pH and temperature.

The sequences of alkaline α-Gals are distinct from their acid isoforms, but are highly homologous with those of raffinose synthase and stachyose synthase [21]. It is interesting to know how these plant specific alkaline α-Gals evolved from their ancestor and acquired their catalytic structural domains. Among six groups, group GAL3 and group AGA3 have less members than others, high-throughput sequencing may find more α-Gals belonging to these two groups. We also found that each α-Gal group contains proteins from both monocots and dicots, and these monocot or dicot α-Gals always cluster into small subgroups, as shown in Fig 3. These results indicated that the ancestor of both acid and alkaline α-Gals existed before monocots and dicots separated.

Four α-Gals highly expressed in cucumber fast-growing fruits, indicating different α-galactosyl residue hydrolysis events occurred in this organ. These events may include RFOs unloading and cell wall loosening and expansion. According to previous studies[9, 21], comparing with the data of the subcellular localizations and expression levels of different α-Gals [9, 21], the RFOs unloading must be carried out by alkaline α-Gals, while the cell wall modification should be performed by CsGAL2 (Fig 4). A more detailed study of assimilate unloading in to cucumber fruits has been conducted in our lab and will be published in another report. The exact role of CsGAL3, a vacuole-localized enzyme, in the fruit development is not clear. Besides fruits, male and female flowers are also sink organs. mRNAs of CsAGA1 and CsAGA2 were predominant α-Gals in male and female flowers respectively, indicating they may catabolize imported RFOs in certain flowers (Fig 4). If it is true, why flowers with different genders choose different α-Gals unloading assimilates is now an enigma.

As a stacyose-translocating species, there should be complicated α-galactosyl residue hydrolysis events during leaf development of cucumber. Like leaves of other plants, at least one acid α-Gal is necessary to loose cell wall for leaf expansion at fast-growing stage. *CsGAL2*, which significantly expressed (Fig 5) and showed a cell-wall localization [9], is mostly perform this function. Besides loading into the phloem, there is a stachyose pool in cucumber leaves for temporary storage (Fig 5) [37]. *CsAGA2*, another high expressed α-Gal in cucumber leaves (Fig 5), may be responsible for the catabolism of accumulated RFOs in cucumber leaves. Several evidences support that alkaline a-Gals catabolize RFOs imported from the phloem in cucurbit sink leaves [14, 38, 39]. The expression of *CsAGA1* increased from leaf 1 to leaf 3 and then declined (Fig 5), the fluctuation pattern is consistent with the sink to source transition process of cucumber leaves (leaf 1 to leaf 3 were sink leaves and leaf 4 to leaf 7 were source leaves) [37], indicating its possible role of RFOs unloading in young cucumber leaves. During leaf senescence, galactolipids and galactoproteins of tonoplast or plastid membrane would be decomposed for reutilization as a source of carbon or energy [6, 40]. In cucumber senescent leaves, *CsGAL3* (vacuole-located) and *CsAGA3* (plastid-located) highly expressed [9], indicating they may play roles in the catabolism of these α-galactosyl-containing compounds. The irregular fluctuation of total acid and alkaline enzyme activities may be due to the complex activity changes of different isoforms during this period. Interestingly, high concentrations of glucose and fructose were found in cucumber senescent leaves, if these monosaccharides are generated from polymer decomposition and could be exported and reutilized is not clear.

Like other fast-growing organs, quick cell wall expansion is absolutely necessary in germination seeds. The mRNA level of *CsGAL2* increased rermarkably during both short and long germinating stages, indicating this isoform fulfills a simmilar role as it in developing fruits and leaves, i.e., loosing cell walls. Another possible role of α-Gals in germinating seeds is hydrolyzing RFOs. In some endospermic seeds, cell-wall acid α-Gals strongly expressed during germination to mobilize galactomannas [41]. While in non- endospermic seeds such as seeds of *Pisum sativum*, which are always lack of galactomannas, RFOs are commonly storage substances [22]. These RFOs could be localized both in the vacuole and the cytoplasm. Thus, both acid and alkaline α-Gals could play roles in RFOs catabolism within certain subcellular compartments [22]. Stachyose is the predominant soluble sugars in mature cucumber seeds [25], although the exact subcellular distribution of these oligosaccharides is unclear. In this study, *CsAGA1* and *CsGAL3* highly expressed during the early and late germinating stage respectively, indicating RFOs in cucumber mature seeds were also comparted into vacuolar and cytoplastic spaces and catabolized by CsGAL3 and CsAGA1 respectively. It is worth noting that CsAGA3, which showed a chloroplast localization, also expressed significantly after cucumber seed imbibition. Thus, the exact distribution of RFOs in seeds during germination needs further investigation.

In summary, we cloned full-length cDNAs of six cucumber α-Gal genes and heterologously expressed them in s/f 9 insect cells. All recombinant proteins revealed acid or alkaline α-Gal activities. These α-Gals showed different substrate specificities, optimum pH and optimum temperatures, indicating their distinct roles during cucumber development. We found six α-Gal genes are widely expressed in different cucumber organs. According to their expression patterns, subcelluar localizations and previous reports, the roles of six cucumber α-Gals were deduced as follows: CsGAL2 mainly exists in fasting-growing organs to loose cell walls; CsGAL3 is localized in vacuoles for catabolizing accumulated RFOs or galactolipids and galactoproteins of the tonoplast membrane; CsAGA1 unloads photoassimilates into sink leaves or male flowers and decomposes cytoplasmic RFOs after seed imbibition; CsAGA2 is reponsible for metabolizing temporarily stored RFOs in leaves; CsAGA3 is localized in pastids and hydrolyzes accumulated RFOs or galactolipids of the plastid membrane during leaf senescence and seed germination; The expression of *CsGAL1* was relatively low in different organs, its role in cucumber development need to further investigation.

## Supporting information

S1 Fig Alignment of acid α-galactosidase cDNA fragments for primers design. Five acid α-galactosidase cDNAs were selected from NCBI and primers for amplifying cDNA fragments were designed in the highly conservative sequence regions. The related acid α-galactosidase used were: *Arabidopsis* AY114020; *Glycine max* U12926; *Lycopersicon esculentum* AF191823; *Pisum sativum* AJ63105; *Phaseolus vulgaris* U12927. *CsGAL* O, the outer of PCR primers; *CsGAL* N, the nested primers.
(JPG)

S2 Fig Alignment of alkaline α-galactosidase cDNA fragments for primers design. Five alkaline α-galactosidase cDNAs were selected from NCBI and primers for amplifying cDNA fragments were designed in the highly conservative sequence regions. The related alkaline α-galactosidase used were: *Arabidopsis* AY090237; *Cucumis melo* AY114164, AY114165; *Lycopersicon esculentum* AF512549; *Persea americana* AJ133148. *CsAGA* O, the outer PCR primers; *CsAGA*, the nested primers.
(JPG)

S3 Fig Fig 1A original image. (JPG)

S4 Fig Fig 1B original image. (JPG)

S1 File (DOCX)
