# Supplementary figures and images for: Characteristics and expression patterns of six α-galactosidases in cucumber (Cucumis sativus L.)
Source: PLoS One. 2021 Jan 12;16(1):e0244714. doi: 10.1371/journal.pone.0244714 (PMC7802950; doi:10.1371/journal.pone.0244714)

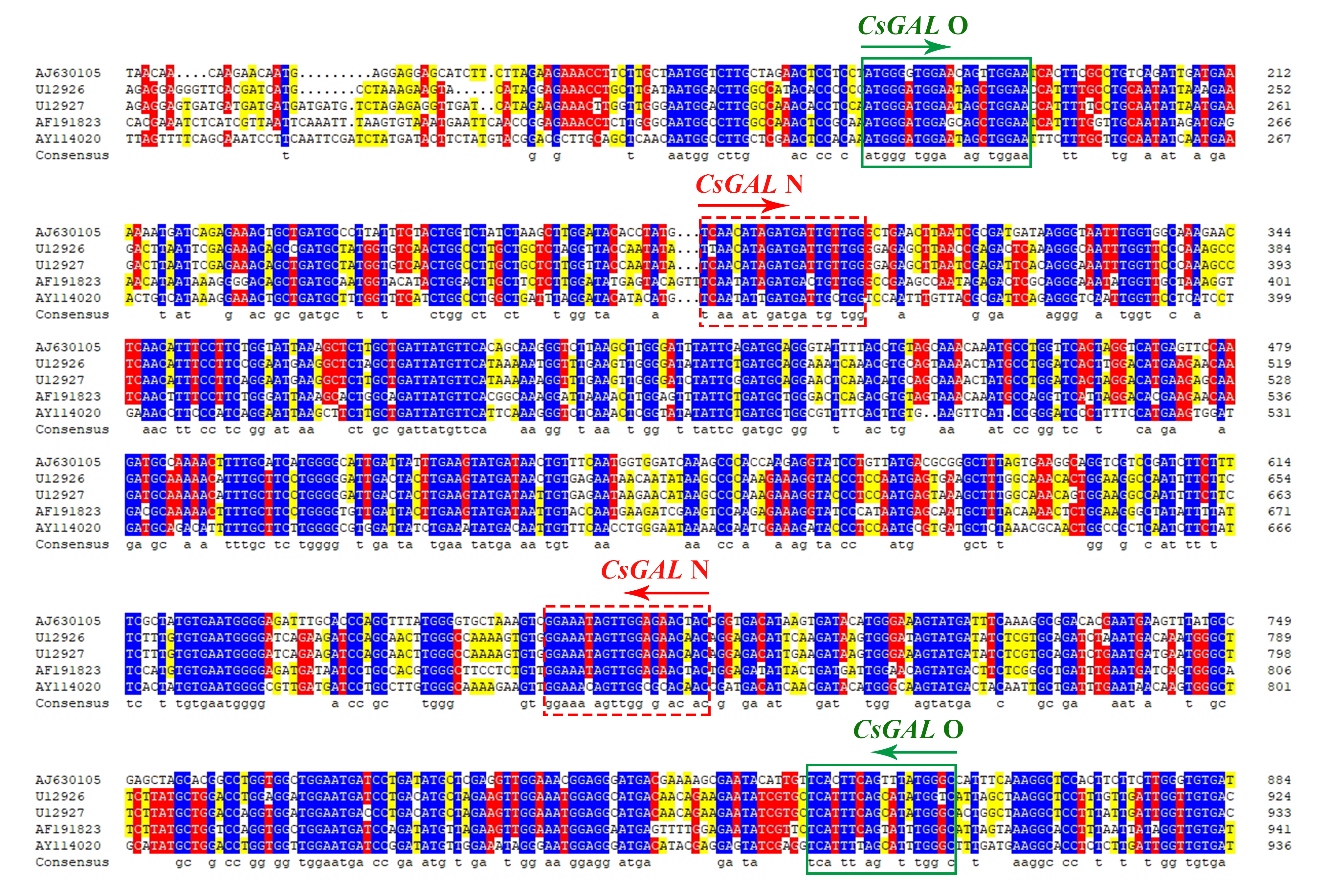

Supplement: S1 Fig — Five acid α-galactosidase cDNAs were selected from NCBI and primers for amplifying cDNA fragments were designed in the highly conservative sequence regions. The related acid α-galactosidase used were: Arabidopsis AY114020; Glycine max U12926; Lycopersicon esculentum AF191823; Pisum sativum AJ63105; Phaseolus vulgaris U12927. CsGAL O, the outer of PCR primers; CsGAL N, the nested primers. (JPG) [file pone.0244714.s001.jpg]

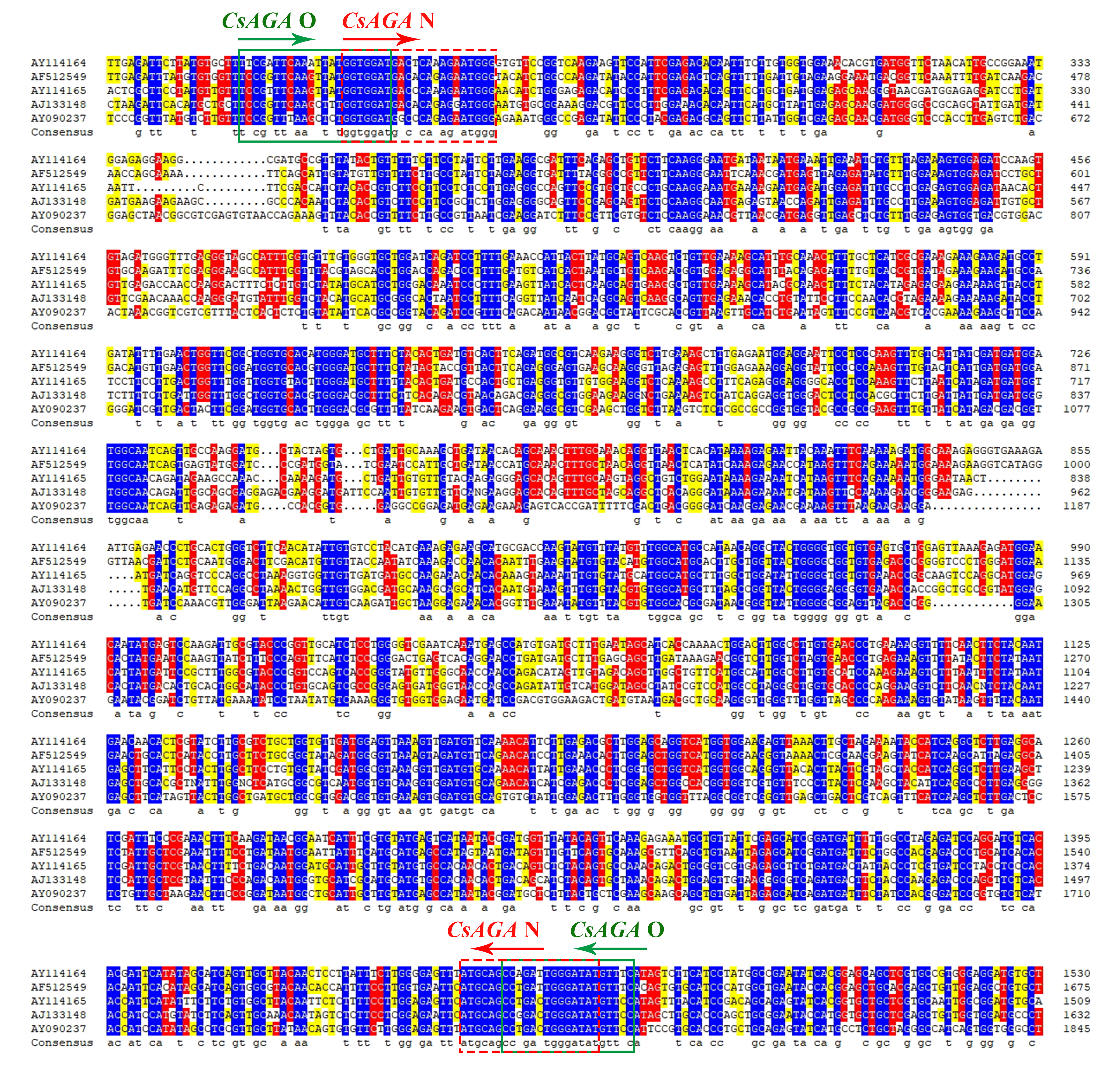

Supplement: S2 Fig — Five alkaline α-galactosidase cDNAs were selected from NCBI and primers for amplifying cDNA fragments were designed in the highly conservative sequence regions. The related alkaline α-galactosidase used were: Arabidopsis AY090237; Cucumis melo AY114164, AY114165; Lycopersicon esculentum AF512549; Persea americana AJ133148. CsAGA O, the outer PCR primers; CsAGA, the nested primers. (JPG) [file pone.0244714.s002.jpg]

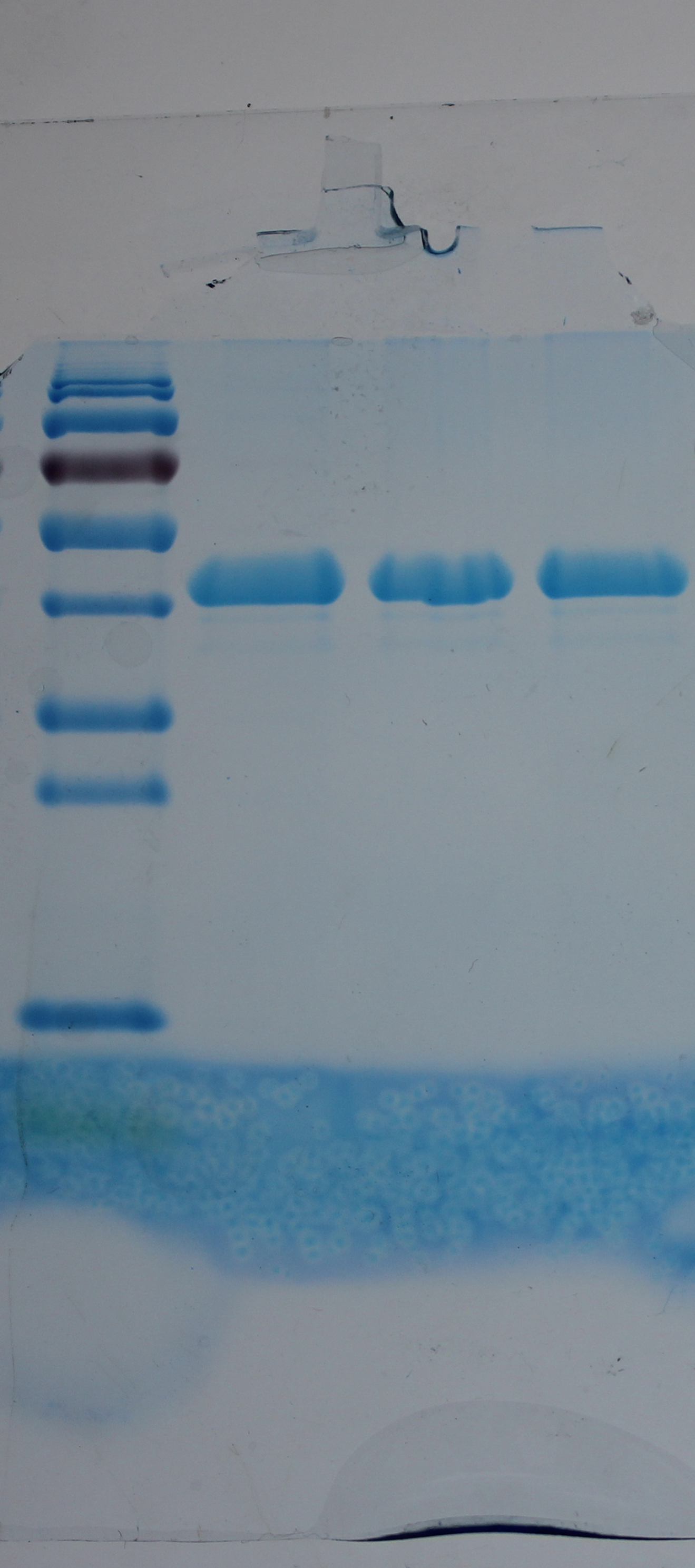

Supplement: S3 Fig — (JPG) [file pone.0244714.s003.jpg]

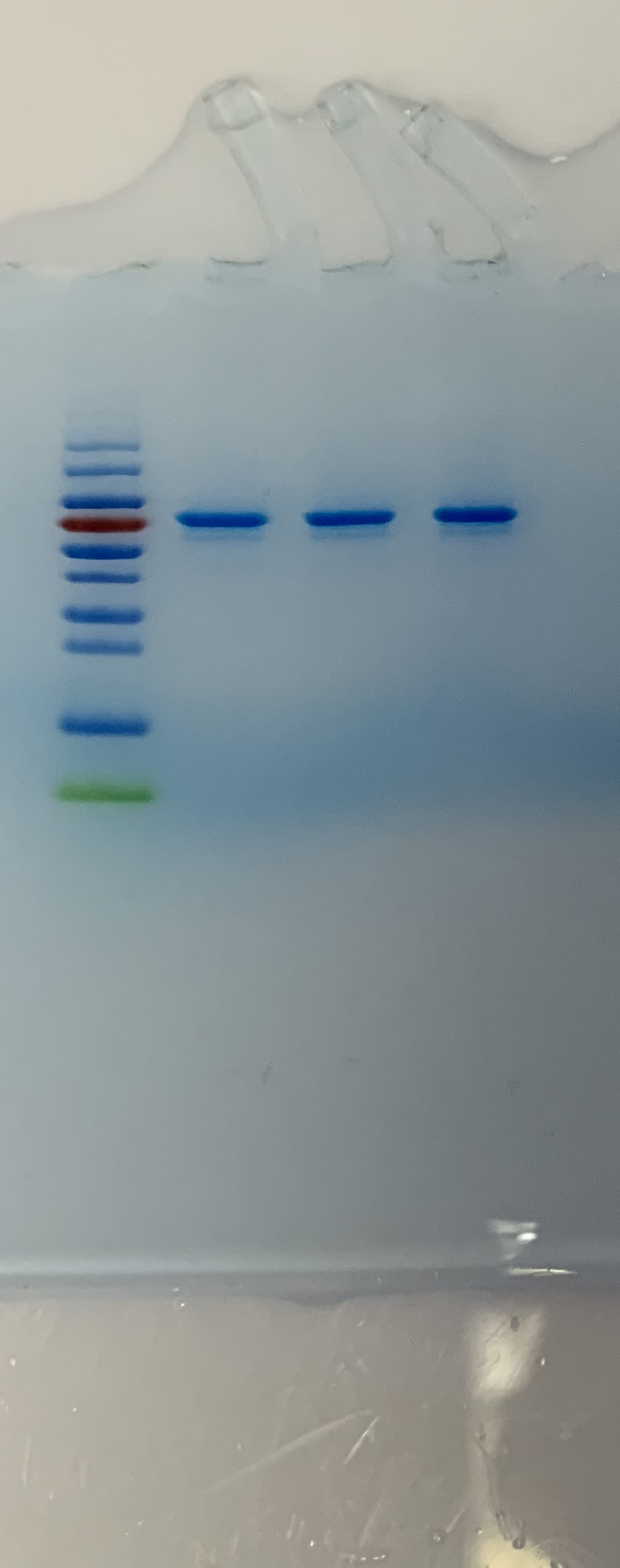

Supplement: S4 Fig — (JPG) [file pone.0244714.s004.jpg]
