# Supplementary material for: Characteristics and expression patterns of six α-galactosidases in cucumber (Cucumis sativus L.)
Source: PLoS One. 2021 Jan 12;16(1):e0244714. doi: 10.1371/journal.pone.0244714 (PMC7802950; doi:10.1371/journal.pone.0244714)
Supplement: S1 File — (DOCX) [file pone.0244714.s005.docx]

**Supplementary Table**

**Supplementary Table S1. Primer sequences for cloning of full-length cDNA of cucumber α-Gals**

| Gene name | Forward primer (5'-3') | Reverse primer (5'-3') | Function |
| --- | --- | --- | --- |
| *CsGAL* F | ATGGGATGGAATAGTTGGAA | GCCCAAATGCTAAAATGA | Conservative fragment acquisition |
| *CsGAL* N | TCAACATAGATGATTGTTGG | GTAGTTCTCCAACTATTTCC |  |
| *CsAGA* F | TCCGTTTCAAGTTATGGTGGAT | GGAACATATCCCAGTCAGGC |  |
| *CsAGA* N | GGTGGATGACTCAAAGAATGGG | ATATCCCAATCAGGCTGCAT |  |
| *CsGAL1* | 3’-RACE GATGATTGTTGGGC TGAGATAGCTCG | 5’-RACE CTCCAACTATTTCC TA ATT TATCGCCCC | Full-length cDNA Clone |
| *CsGAL2* | 3’-RACE TAGATGATTGTTGG GCTGAACTTGAC | 5’-RACE AGGACCTGCCAAA CCTCGAGGTCTCC |  |
| *CsGAL3* | 3’-RACE TGCCTCCATTACCAA CTTCCAACATATCT | 5’-RACE GACAGACGACATTA ATAGATACATGGGCAA |  |
| *CsAGA1* | 3’-RACE GTGTAGCAGTGGAG TCGGAGGAGGTAGAG | 5’-RACE CCTCAAACCCATCT ACA CTTGGATCTCCG |  |
| *CsAGA2* | 3’-RACE GGACATCACCTCAT ACATCTCGTTTGCC | 5’-RACE TCGACGATTCCACC AT GACCAGCACC |  |
| *CsAGA3* | 3’-RACE TGATGAACCCTAAG AATGTTTACAAATTTTA | 5’-RACE CTGGAACTTCGAATT CTCTCTGATTGCGGTC |  |

Note: F: Note: O and F: the outer primers and the nested primers of Nested PCR, 3’-RACE: 3’-RACE primer, 5’-RACE: 5’-RACE primer

**Supplementary Table S2. Primer sequences for construction of insect cell expression vector**

| Gene Names | Vectors | Forward primer (5'-3') | Reverse primer (5'-3') |
| --- | --- | --- | --- |
| *CsGAL1* | pAcHLT-A | AAC AGG CCT ACGGTTGGTGCTGG | ATT GAGCT C TCATAGTTCAATTCT |
| *CsGAL2* | pAcHLT-A | TCA G AATTC ACGGTCACACCGAA | AAC CCC GGG CTAAACTTCAATTTC |
| *CsGAL3* | pAcHLT-A | AGA AGG CCT ACGATCAAACCAGCT | CCA GAGCT C TCATAATTCAATT |
| *CsAGA1* | pAcHLT-A | ATA AGG CCT GAGTGCCGGAGTTAC | TAC GAGCT C TCAAGAAATTGGCTT |
| *CsAGA2* | pAcHLT-A | TCA AGG CCT GCTCTTCCCCCATCT | TTT GAGCT C TTAGTGAGGTGTGAGG |
| *CsAGA3* | pAcHLT-A | CTA G AATTC GCGAACAGGATAAG | ATA GAGCT C TTACATTTCAGCACG |

Note: The restriction sites introduced in primers were highlighted with underlines, *Eco*RI, G AATTC, *Sac*I: GAGCT C, *Sma*I, CCC GGG, *Stu*I: AGG CCT.

**Supplementary Table S3 Possible functions and papers reported of α-Gals**

| Groups | Plant sources | Accession number | Functional verification | Expression position or physiological process involved | Reference |
| --- | --- | --- | --- | --- | --- |
| *GAL1* | *Arabidopsis* | NP_196455 | No | / | / |
|  | *Arabidopsis* | CAC08337 | No | / | / |
|  | *Carica papaya* | AAP04002 | Yes | Fruit ripening | [[1](#_ENREF_1)] |
|  | *Cucumis sativus* | ABC55266 | Yes | Leaves, fruits, roots and flowers | [[2](#_ENREF_2)] |
|  | *Hordeum vulgare* | KAE8783913 | No | Natural senescence and dark induced senescence of leaves | [[3](#_ENREF_3)] |
|  | *Oryza sativa* | AAG13536 | No | / | / |
|  | *Pisum sativum* | CAF34023 | No | Seeds | / |
| *GAL2* | *Arabidopsis* | NP_001031855 | No | Binding to cell wall | [[4](#_ENREF_4)] |
|  | *Cyamopsis tetragonoloba* | CAA32772 | Yes | Aleurone cells of germinated seeds | [[5](#_ENREF_5)] |
|  | *Coffea arabica* | AAA33022 | Yes | Endosperm development | [[6](#_ENREF_6)] |
|  | *Cocos nucifera* | AIL28756 | Yes | Endosperm | / |
|  | *Cucumis sativus* | ABC88435 | Yes | Leaves, fruits, roots and flowers | [[2](#_ENREF_2)] |
|  | *Glycine max* | AAA73963 | No | Seedlings | / |
|  | *Helianthus annuus* | BAC66445 | No | Seeds | / |
|  | *Hordeum vulgare* | KAE8783909 | No | Leaf development and dark induced senescence | [[4](#_ENREF_4)] |
|  | *Lycopersicon esculentum* | AAF04591 | No | Seed development and germination | [[7](#_ENREF_7)] |
|  | *Nicotiana tabacum* | AEB98600 | Yes | Nectar | [[8](#_ENREF_8)] |
|  | *Oryza sativa* | BAB12570 | Yes | Cell in suspension culture | [[9](#_ENREF_9)] |
|  | *Petunia hybrida* | AAQ82455 | No | Normal temperature after low temperature stress | [[10](#_ENREF_10)] |
|  | *Oryza sativa* | BAC55816 | No | Vacuole | [[11](#_ENREF_11)] |
|  | *Phaseolus vulgaris* | AAA73964 | No | Seedlings | / |
|  | *Vitis viniferaL.* | XP_002268711 | No | / | / |
| *GAL3* | *Arabidopsis* | AAM45068 | No | / | / |
|  | *Cucumis sativus* | AEQ94270 | Yes | Leaves, fruits, roots, flowers and callus | [[12](#_ENREF_12)] |
|  | *Oryza sativa* | BAD31216 | Yes | Seedlings growing for 2 weeks | [[11](#_ENREF_11)] |
| *AGA1* | *Arabidopsis* | AAO42886 | No | / | / |
|  | *Arabidopsis* | NP_680552 | No | / | / |
|  | *Oryza sativa* | AAL65392 | Yes | Dark induced leaf senescence | [[13](#_ENREF_13)] |
|  | *Zea mays* | AAQ07253 | Yes | Seeds germination | [[14](#_ENREF_14)] |
|  | *Lycopersicon esculentum* | AAN32954 | Yes | Seeds | [[15](#_ENREF_15)] |
|  | *Cucumis melo* | AAM75139 | Yes | Fruit, RFOs unload | [[15](#_ENREF_15)] |
|  | *Cucumis sativus* | AAZ81424 | Yes | Leaves, fruits, roots and flowers | [[2](#_ENREF_2)] |
| *AGA2* | *Persea american* | CAB77245 | No | Fruits during storage | / |
|  | *Arabidopsis* | CAB66109 | No | / | / |
|  | *Brassica oleraceae* | X79330 | No | Root tips of freshly germinated seeds | / |
|  | *Cucumis melo* | AAM75140 | Yes | Fruit | [[15](#_ENREF_15)] |
|  | *Cucumis sativus* | ABD52008 | Yes | Leaves, fruits, roots and flowers | [[2](#_ENREF_2)] |
|  | *Hordeum vulgare* | AAA32975 | No | Embryo of seeds germinating for 24 hours | / |
|  | *Oryza sativa* | XP_477103 | No | / | / |
|  | *Pisum sativum* | ABR19752 | Yes | Seeds | [[16](#_ENREF_16)] |
|  | *Tetragonia tetragonioides* | BAG74565 | Yes | Drought stress | [[17](#_ENREF_17)] |
|  | *Vitis viniferaL.* | ACD39775 | Yes | Osmotic stress | [[18](#_ENREF_18)] |
|  | *Zea mays* | AAQ07251 | Yes | Seed germination, induced by temperature stress | [[14](#_ENREF_14)] |
| *AGA3* | *Zea mays* | AAQ07252 | No | Seeds germination | [[14](#_ENREF_14)] |
|  | *Arabidopsis* | AAL90901 | No | Dark induced leaf senescence | [[19](#_ENREF_19)] |
|  | *Cucumis sativus* | AFA34435 | No | / | [[20](#_ENREF_20)] |

**Supplementary Table S4. Primer sequences for qRT-PCR of cucumber α-Gals**

| Gene name | Forward primer (5'-3') | Reverse primer (5'-3') |
| --- | --- | --- |
| *18S* r*RNA* | TCTATAGCCTTGGCCGACAG | TACAAAGGGCAGGGACGTAG |
| *CsGAL1* | TTACGAACACAGAAGTCATTGCAGT | GTCCCATTGTGCCGAGATTGC |
| *CsGAL2* | CTGAGTTTGATTCTGCTTCCTCTAGG | CTGATATCCTAATGCAGCAAGTCCG |
| *CsGAL3* | TGATACCTCCAAGTACGGCATAC | CGTACACATAACCTAACTCAGCC |
| *CsAGA1* | GAGAATCCCGACACAATCACAGG | TCGATGCATCTTGTGGTAGGTAAAC |
| *CsAGA2* | AGTTCCTGTTACCCTCAAAGTTTTAGAAT | GACCACTTGGATATCGACTTGCTC |
| *CsAGA*3 | CCGCAATCAGAGAGAATTCGAAGTT | GATGATCCGTATTCCTCCATGTCCT |

Supplementary Reference

1. Soh CP, Ali ZM, Lazan H. Characterisation of an alpha-galactosidase with potential relevance to ripening related texture changes. Phytochemistry. 2006;67(3):242-54. pmid: 16325871.

2. Xu XF. Cloning and expression analysis of α-galactosidases in cucumber (*Cucumis sativus* L.). China: Yangzhou University; 2006.

3. Chrost B, Krupinska K. Genes with homologies to known alpha-galactosidases are expressed during senescence of barley leaves. Physiol Plant. 2000;110(1):111-9. wos:000089349700015.

4. Chrost B, Kolukisaoglu U, Schulz B, Krupinska K. An alpha-galactosidase with an essential function during leaf development. Planta. 2007;225(2):311-20. pmid: 16845526.

5. Hughes SG, Overbeeke N, Robinson S, Pollock K, Smeets FL. Messenger RNA from isolated aleurone cells directs the synthesis of an alpha-galactosidase found in the endosperm during germination of guar (Cyamopsis tetragonaloba) seed. Plant Mol Biol. 1988;11(6):783-9. pmid: 24272628.

6. Marraccini P, Rogers WJ, Caillet V, Deshayes A, Granato D, Lausanne F, et al. Biochemical and molecular characterization of alpha-D-galactosidase from coffee beans. Plant Physiol Biochem. 2005;43(10-11):909-20. pmid: 16310367 .

7. Feurtado JA, Banik M, Bewley JD. The cloning and characterization of alpha-galactosidase present during and following germination of tomato (*Lycopersicon esculentum* Mill.) seed. J Exp Bot. 2001;52(359):1239-49. pmid: 11432942.

8. Zha HG, Flowers VL, Yang M, Chen LY, Sun H. Acidic alpha-galactosidase is the most abundant nectarin in floral nectar of common tobacco (*Nicotiana tabacum*). Ann Bot. 2012;109(4):735-45. pmid: 22271925.

9. Kim WD, Kobayashi O, Kaneko S, Sakakibara Y, Park GG, Kusakabe I, et al. Alpha-galactosidase from cultured rice (*Oryza sativa* L. var. Nipponbare) cells. Phytochemistry. 2002;61(6):621-30. pmid: 12423882.

10. Pennycooke JC, Vepachedu R, Stushnoff C, Jones ML. Expression of an alpha-galactosidase gene in Petunia is upregulated during low-temperature deacclimation. J Am Soc Hort Sci. 2004;129(4):491-6. pmid: 14500789.

11. Li SH, Kim WD, Kaneko S, Prema PA, Nakajima M, Kobayashi H. Expression of rice (*Oryza sativa* L. var. Nipponbare) alpha-galactosidase genes in Escherichia coli and characterization. Biosci Biotech Bioch. 2007;71(2):520-6. pmid: 17284836.

12. Wang CL, Zhang ZP, Miao MM. SNF1-related protein kinase (SnRK) 1 involved in the regulation of raffinose family oligosaccharide metabolism in cucumber (*Cucumis sativus* L.) calli. J Plant Growth Regul. 2016;35(3):851-64. wos: 000387794900023.

13. Lee RH, Lin MC, Chen SCG. A novel alkaline alpha-galactosidase gene is involved in rice leaf senescence. Plant Mol Biol. 2004;55(2):281-95. PMID: 15604681.

14. Zhao TY, Corum JW, Mullen J, Meeley RB, Helentjaris T, Martin D, et al. An alkaline alpha-galactosidase transcript is present in maize seeds and cultured embryo cells, and accumulates during stress. Seed Sci Res. 2006;16(2):107-21. wos: 000237234400003.

15. Carmi N, Zhang G, Petreikov M, Gao Z, Eyal Y, Granot D, et al. Cloning and functional expression of alkaline alpha-galactosidase from melon fruit: similarity to plant SIP proteins uncovers a novel family of plant glycosyl hydrolases. Plant J. 2003;33(1):97-106. pmid: 12943544.

16. Blochl A, Peterbauer T, Hofmann J, Richter A. Enzymatic breakdown of raffinose oligosaccharides in pea seeds. Planta. 2008;228(1):99-110. pmid: 18335235.

17. Hara M, Tokunaga K, Kuboi T. Isolation of a drought-responsive alkaline alpha-galactosidase gene from New Zealand spinach. Plant Biotechnol. 2008;25(5):497-501. wos: 000262640200011.

18. Daldoul S, Toumi I, Reustle GM, Krczal G, Ghorbel A, Mliki A, et al. Molecular cloning and characterisation of a cDNA encoding a putative alkaline alpha-galactosidase from grapevine (*Vitis vinifera* L.) that is differentially expressed under osmotic stress. Acta Physiol Plant. 2012;34(3):891-903. wos: 000302811000007.

19. Fujiki Y, Yoshikawa Y, Sato T, Inada N, Ito M, Nishida I, et al. Dark-inducible genes from Arabidopsis thaliana are associated with leaf senescence and repressed by sugars. Physiol Plant. 2001;111(3):345-52. pmid: 11240919.

20. Zhang ZP, Deng YK, Song XX, Miao MM. Trehalose-6-phosphate and SNF1-related protein kinase 1 are involved in the first-fruit inhibition of cucumber. J Plant Physiol. 2015;177:110-20. pmid: 25723473.
